# Altered Brain Activation in Early Drug-Naive Parkinson's Disease during Heat Pain Stimuli: An fMRI Study

**DOI:** 10.1155/2015/273019

**Published:** 2015-01-05

**Authors:** Ying Tan, Juan Tan, Cheng Luo, Wenjuan Cui, Hui He, Yi Bin, Jiayan Deng, Rui Tan, Wenrong Tan, Tao Liu, Nanlin Zeng, Ruhui Xiao, Dezhong Yao, Xiaoming Wang

**Affiliations:** ^1^The Key Laboratory for NeuroInformation of Ministry of Education, University of Electronic Science and Technology of China, Chengdu 610054, China; ^2^School of Computer Science and Technology, Southwest University for Nationalities, Chengdu 610041, China; ^3^Neurology Department, Affiliated Hospital of North Sichuan Medical College, North Sichuan Medical College, Nanchong 637000, China; ^4^School of Life and Science Engineering, Southwest Jiaotong University, Chengdu 610031, China

## Abstract

Parkinson's disease (PD) is a progressive neurodegenerative disease characterized by motor and nonmotor signs and symptoms. To date, many studies of PD have focused on its cardinal motor symptoms. To study the nonmotor signs of early PD, we investigated the reactions solicited by heat pain stimuli in early untreated PD patients without pain using fMRI. The activation patterns of contact heat stimuli (51°C) were assessed in 14 patients and 17 age- and sex-matched healthy controls. Patients with PD showed significant decreases in activation of the superior temporal gyrus (STG) and insula compared with controls. In addition, a significant relationship between activation of the insula and STG and the pain scores was observed in healthy controls but not in PD. This study provided further support that the insula and STG are important parts of the somatosensory circuitry recruited during the period of pain. The hypoactivity of the STG and insula in PD implied that functions including affective, cognitive, and sensory-discriminative processes, which are associated with the insula and STG, were disturbed. This finding supports the view that leaving early PD untreated could be tied directly to central nervous system dysfunction.

## 1. Introduction

Parkinson's disease (PD), the second most common neurodegenerative disease, belongs to a group of conditions called motor system disorders. Although difficulties with movement are prominent in PD patients, the nonmotor signs and symptoms are likewise frequent in these patients. Nonmotor symptoms include cognitive impairment, sleep disorders, dysautonomia, and pain. Pain is one of the most frequent nonmotor symptoms that affect PD patients. Several recent epidemiological studies have found that 70–80% of patients with PD suffer from pain sensations [1].

Pain in PD patients is thought to result from deficient sensory information. Previous studies have indicated that patients with PD exhibit numerous abnormalities in somatosensory perception [2–5]. However, findings are inconsistent across studies involving various treatments. During nociceptive stimulation before levodopa challenge (the “OFF” condition), Brefel-Courbon et al. [6] found that PD patients experiencing pain displayed lower pain activation in the right prefrontal cortex and posterior insula, as well as higher pain activation in the right anterior cingulate cortex than pain-free patients; moreover, the same research team [7] reported a significant increase in pain-induced activation of the right insula and prefrontal and left anterior cingulate cortices in PD patients compared with controls. Furthermore, the brain area activated by pain is described differently in studies using subthalamic nucleus deep-brain stimulation (STN-DBS) [8–11], an emerging interventional therapy for motor complications in patients with PD.

The aforementioned studies demonstrate that some problems remain unclear. For example, the same treatment sometimes produced different results, which we inferred might be due to differences in treatment duration or measurement method. Although drugs such as levodopa and STN-DBS have been demonstrated to be valid treatments for patients with PD, their mechanisms remain unclear. Thus, this work focused on drug-naive early PD patients and explored a neural mechanism that could be used for clinical diagnosis.

Little is known about the sensory symptoms of drug-naive early PD patients and their mechanisms. Luo et al. [12], using resting-state functional connectivity magnetic resonance imaging (rs-fMRI), revealed that early-stage drug-naive PD patients exhibit reduced functional connectivity in mesolimbic-striatal and corticostriatal loops relative to healthy controls. However, this study did not exclude PD patients with pain and did not measure pain symptoms of PD patients using quantitative methods. Event-related fMRI, which has been widely used in cognitive studies and for clinical applications such as determining epileptic location [13], provides a methodology by which alterations correlated with pain stimuli in PD patients are uncovered.

The current study aimed to explore the functional reorganization of pain-related pathways in early drug-naive PD patients without pain through whole-brain blood oxygenation of level-dependent (BOLD) fMRI during pain stimulus using a contact heat-evoked potential stimulator (CHEPS). Moreover, to understand how functional activation causes changes in the pain matrix, we investigated the relationship between pain and brain activation.

## 2. Materials and Methods

### 2.1. Subjects

A total of 14 right-handed patients with a diagnosis of idiopathic PD were enrolled in the MRI study at the Neurology Department of the Institute of Neurological Disease, North Sichuan Medical College, China. The diagnosis of early PD was made according to the UK PD Society Brain Bank diagnostic criteria [14] by experienced neurologists at the Affiliated Hospital of North Sichuan Medical College, based on standard physical and neurological examinations, laboratory tests, and MRI scans, and all of the patients were required not to have been treated with anti-Parkinson drugs prior to the initial visit (i.e., to be drug-naive). Patients with moderate-to-severe head tremor, a history of head injury, stroke or another neurologic disease, or any disorder that interfered with the assessment of the manifestation of PD were excluded.

A total of 17 right-handed, age- and sex-matched healthy participants were selected for the control group with no history or presentation of any Diagnostic and Statistical Manual of Mental Diseases Axis-V diagnosis, with no neurologic illness, and, as assessed by clinical evaluations and medical records, with no brain lesion on traditional MRI. All subjects underwent an MRI examination prior to entering the present study to exclude subjects with any relevant structural abnormalities. None of the patients had T2-weighted hyperintensities in the deep white matter. The study was performed according to the standards set by the Declaration of Helsinki and was approved by the Ethics Committee of the Affiliated Hospital of North Sichuan Medical College; written informed consent was obtained for all subjects.

### 2.2. Experimental Procedures

Contact heat stimulation (CHS) was delivered to the skin of the right dorsal forearm using CHEPS (Medoc Ltd., Ramat Yishai, Israel), a heat-foil with rapid rising time at high temperatures (up to 70°C/S) developed to study pain activation related to thermal and nociceptive pathways. CHEPS has a probe with (at one end) a thermode area of 572.5 mm^2^ and a heating thermofoil (Minco Products, Inc., Minneapolis, MN) covered with a 25-*μ*m layer of thermoconductive plastic. The experiments included the 51°C thermal stimulus. All stimuli were initiated from a baseline temperature of 32°C increased to a target temperature 51°C applied by computer-controlled brief radiant pulses. Twenty stimuli were included in each session. The interval of stimuli was manipulated within 16, 20, or 24 seconds at random to avoid habituation. The subjective perception of the experimental pain was recalled and assessed by the subjects, who were asked to verbally rate the intensity of the experimental pain by means of a visual analog scale (VAS) ranging from zero (“no pain”) to ten (“worst pain imaginable”) once outside the scanner. Finally, warm sense thresholds (WST) and heat-induced pain thresholds (HPT) were measured by quantitative sensory testing (QST) for each subject.

### 2.3. MRI Data Acquisition

All fMRI data were acquired using gradient echo-planar imaging (EPI) sequences in a 3 T MRI scanner (EXCITE, GE Milwaukee) with a 32-channel phased array head coil. The imaging parameters were as follows: thickness, 4 mm (no gap); TR = 2,000 ms; TE = 30 ms; FOV = 240 mm × 240 mm; flip angle = 90°; and matrix = 64 × 64. Two hundred and five volumes (35 slices per volume) were obtained during 410 seconds of an fMRI session. To ensure steady-state longitudinal magnetization, the first five volumes were excluded. During data acquisition, participants were instructed to relax with their eyes closed without falling asleep [15]. Anatomical T1-weighted images were acquired using a three-dimensional (3D) spoiled gradient recalled (SPGR) sequence, generating 156 axial slices (thickness: 1 mm (no gap), TR = 8.2 ms, TE = 3.2 ms, FOV = 240 mm × 240 mm, flip angle = 12°, and matrix = 256 × 256).

### 2.4. Preprocessing

Preprocessing of the fMRI data was performed using the SPM8 software package (statistical parametric mapping, http://www.fil.ion.ucl.ac.uk/spm/). Each dataset was realigned. The time was corrected to reflect differences in image acquisition time between slices, followed by normalization to transform the image to match the template supplied with SPM (MNI 152) [16]. Then, the image was resampled to 3 × 3 × 3 mm^3^. Finally, images were smoothed using an isotropic Gaussian kernel (8 mm full width at half maximum).

### 2.5. Statistics Tests

For each subject, data were analyzed on a pixel level using the general linear model (first level). In this model, the time course produced by a time pulse function of stimuli convolved with a canonical hemodynamic response function was used as an interesting regressor to analyze task-dependent activation. Meanwhile, the six parameters of head motion were designated as noninteresting regressions. Then, a one-sample *t*-test was used to determine brain activity within each group (*P* < 0.05, FDR corrected, cluster size > 600 mm^3^ (23 adjacent voxels)), whereas a two-sample *t*-test model (*P* < 0.005, uncorrected, cluster size > 600 mm^3^) was used for between-group comparisons to control for age and gender effects.

### 2.6. Correlations with Clinical Features

To investigate the relationship between the altered functional variables and the clinical variables, we chose the significantly altered boxes as the region of interest (ROI) and calculated the mean value of the regression coefficients (*β* value). We obtained the correlation between the mean *β* value and the subjective scores of pain (VAS and QST) using partial correlation analysis with covariates of age and gender.

## 3. Results

### 3.1. Demographic and Clinical Characteristics

All the patients were assessed with unified Parkinson's disease rating scale (UPDRS) and the mini-mental state examination (MMSE). The pain threshold of each subject was assessed by means of VAS and QST once outside the scanner. The demographics and clinical characteristics of subjects are shown in Table 1.

### 3.2. Activation Map within Groups

**Figure 1:**
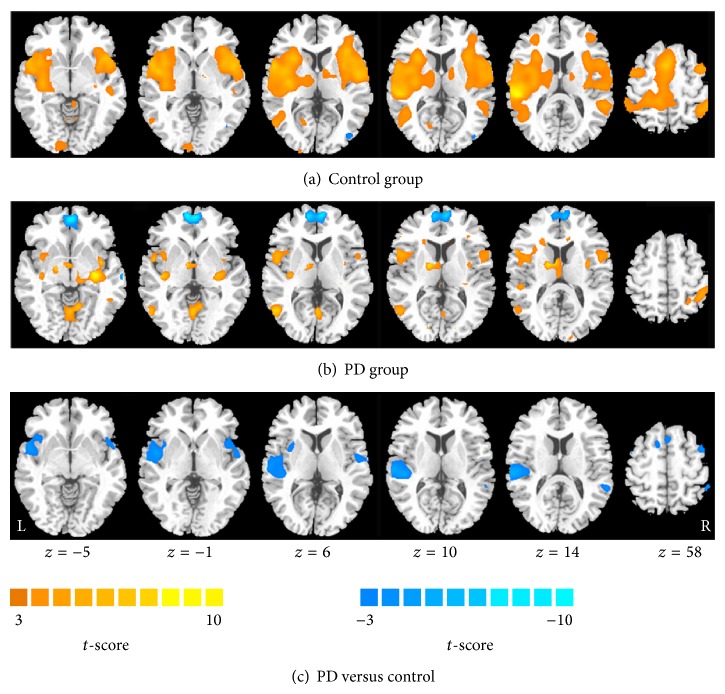
Within-group activation maps in response to heat stimulation in the control group (a) and the PD group (b). Activation is represented as hot and deactivation as cool. Row (c) shows the difference of activation between groups. Cool color represents the decreased activation in patients with PD in contrast to healthy controls.

Within the control group, the bilateral STG, insula, supramarginal gyrus, putamen, contralateral superior parietal lobule, supplementary motor area, cingulate cortex, rolandic operculum, primary somatosensory cortex (S1), calcarine fissure, and ipsilateral frontal gyrus of the cerebellum were strongly activated in response to 51°C heat stimuli (Table 2 and Figure 1(a)). In the PD group, however, the activated regions and intensities were dramatically less than those of the control group (Table 3 and Figure 1(b)), such as STG, insula, S1, and putamen. Furthermore, the bilateral medial frontal lobe had obviously been deactivated.

### 3.3. Difference of Activation between Groups

During heat stimulation, patients with PD showed decreased activation of the bilateral STG, insula and ipsilateral temporal pole, and middle temporal gyrus (Figure 1(c) and Table 4) under the painful thermal stimulation.

### 3.4. Relationship to Clinical Features

**Figure 2:**
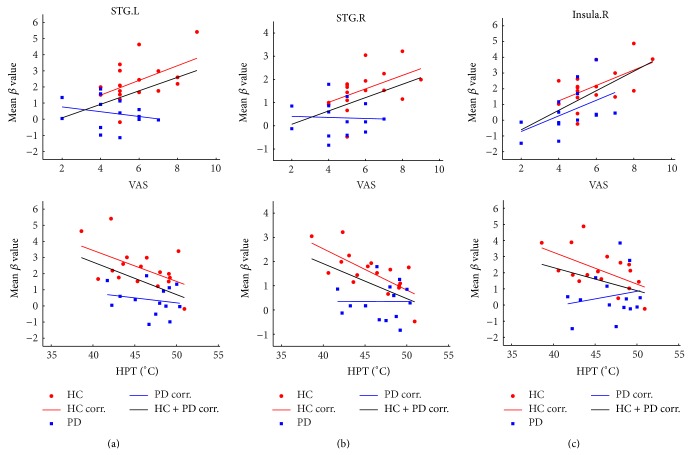
Linear partial correlation coefficients between the mean values of *β* (for left STG, right STG, and right insula) and indices (VAS, QST), controlling for the effects of age and gender. The significance is provided in Table 5.

Linear partial correlation coefficients between the mean values of *β* values in ROIs (insula, STG, and temporal pole) and indices (VAS, QST), when controlling for the effect of age and gender, were observed (Figure 2 and Table 5). In the relationship analysis of VAS and mean *β* value, positive correlations in the bilateral STG and right insula cortex (INS.R, stimuli-ipsilateral side) were observed in the control group, whereas only the stimuli-ipsilateral insula cortex (right) had a positive relationship with the VAS score in the PD group. No correlation was discovered in the stimuli-contralateral insula cortex (left) in either group. In the relationship analysis of QST (including HPT and WST) and mean *β* value, a negative correlation was noteworthy in the bilateral STG and INS.R in the control group, whereas no correlation was found in the PD group. No correlation was found between WST score and the fMRI signal in either group.

## 4. Discussion

In this fMRI study, patients with PD demonstrated significantly attenuated activation of the STG and insula during noxious heat stimulation (51°C). Furthermore, pain was evaluated using the VAS scale, and thermal thresholds were evaluated using QST. After the heat pain (51°C) stimulus, significant alteration between two groups was investigated.

### 4.1. Altered Activation in the STG

Activity in the STG has been reported in many pain-induced studies [17–20], although it is generally involved in auditory processing, language, and social cognition. However, the STG has largely been ignored in pain imaging studies, likely in part because the link between STG function and pain is not obvious and because many investigators pay more attention to their regions of interest, such as the insula.

In our study, contralateral (relative to the stimuli) STG was more highly activated in HC during the thermal pain task, whereas it was hypoactive in the PD patients. There were three reasons associated with this finding. First, the STG was incorporated into the somatosensory circuitry. Our study suggests that the STG would be associated with pain perception and negative emotion. The STG perceives the increased sense of negative emotion, which includes the unpleasant feelings produced by pain. The STG also likely plays a role in the efference copy function mediated by the putamen, which is involved in pain processing [21, 22]. In addition, the STG was associated with the anticipation of pain [23, 24]. Second, the PD patient group showed decreased functional connectivity in the STG [12, 25]. Executive dysfunction in PD patients may be linked to dopaminergic deficits in the STG [26]. Third, the loss of gray matter in the STG region, which is responsible for the onset of PD, is consistent with the earlier findings reported in medical studies [27]. The thickness of the STG is correlated with the degree of activation and cognitive dysfunction, which is partly confirmed in Pagonabarraga's paper [28]. In addition, the activation of the STG was positively correlated with VAS ratings and negatively correlated with HPT ratings under pain stimulation in the HC group. There was no activation of the STG correlated with VAS and QST ratings in the PD group. Our study suggests that the STG is involved in the processing of pain-related unpleasantness and is negatively affected in patients with PD.

### 4.2. Altered Activation of the Insula

The insula plays a key role in processing noxious and innocuous thermal stimuli [29]. The insula was functionally connected to several brain areas, thereby forming a pain matrix, which involved different areas involved in pain perception, including S1 and the cingulate gyrus, prefrontal cortex, and parietal association cortices. Meanwhile, the anterior insula was more strongly functionally connected to areas known for affective and salience processing [30–32], whereas the posterior insula was more strongly connected with areas known for sensory-discriminative processing of noxious and somatosensory stimuli [6, 33]. Our results revealed that the insula is also highly activated, which is accompanied by the activation of the basal ganglia and many other cortical regions including the frontal, temporal, parietal, and cingulate cortices.

PD, beyond its hallmark motor symptoms, causes variable degrees of cognitive impairment in a high percentage of patients [34]. The prevalence of cognitive deficits in untreated, newly diagnosed patients is between 19 and 24% [34]. PD patients with pain illustrated lower activation in the bilateral posterior insula resulting from painful stimuli [6]. The anterior insula, which effects pathological processes, could disrupt both cognitive and social functions, and the posterior insula is linked to bodily sense of awareness. Therefore, we infer that the hypoactivity of the insula in the PD group may be experienced subjectively as a decrease in the thermal stimulus produced by the combination of anterior insula and posterior insula. Previous studies [30] have proven that perceived thermal intensity was well correlated with activation of the ipsilateral anterior insula. Consistent with this study, we found a correlation of activation with VAS and HPT in HC in the right insula but not in the left insula. Taken together, our finding supports that the insula was the most impaired region during perception of the heat stimulus in the PD group. Furthermore, the anterior insula, which has an important effect on affective, cognitive, and sensory-discriminative processes, was interrupted in the PD group.

## 5. Conclusion

Despite the fact that the diagnosis of Parkinson's disease is predominantly based on clinical signs, neuroimaging offers great promise for the study of disease pathogenesis and evolution, particularly for studying brain functional changes during the early stage of disease. We provided evidence of abnormal activation in early drug-naive PD patients during a heat pain stimulus. In particular, we report observably decreased activation upon nociceptive pain (51°C) in PD subjects without pain symptoms. This result should contribute to the future clinical treatment of pain in PD patients. Furthermore, this finding supports the crucial role of the insula and STG in pain processing, as the insula is the most impaired region during perception of the heat stimulus in the PD group. In summary, our findings add to the growing evidence that early PD could be related directly to central nervous system dysfunction involving pain.

## Figures and Tables

**Table 1:** Demographics, clinical characteristics, and sensory thresholds.

	PD (*n* = 14)	CON (*n* = 17)	*P* value
	Means ± SD	Means ± SD	
Age (years)	62.79 ± 4.59	61.35 ± 4.27	0.376
Gender^*^	11 M/3 F	13 M/4 F	0.889
Disease duration (years)	2.46 ± 1.43	—	—
UPDRS score			
Part I—mood/cognition	2.93 ± 1.14	—	—
Part II—activities of daily living	9.93 ± 2.27	—	—
Part III—motor examination	21.79 ± 5.66	—	—
Part IV—complications of therapy	0	—	—
MMSE score	23.93 ± 2.13	—	—
VAS	4.77 ± 1.30	5.69 ± 1.25	0.064
WST	37.01 ± 1.44	35.74 ± 1.39	0.022
HPT	47.74 ± 2.09	45.33 ± 3.44	0.042

Note: ^*^Chi-square test.

M, male; F, female; UPDRS, unified Parkinson's disease rating scale; MMSE, mini-mental state exam; VAS, visual analog scale; WST, warm sense thresholds; HPT, heat-induced pain thresholds.

**Table 2:** Activation in response to heat stimulation in the control group.

Region	Control group						
	Laterality	BA	*Z*	MNI coordinates (mm)			Cluster
			Score	*x*	*y*	*z*	(Voxels)
Superior temporal gyrus	L	42	5.94	−54	−30	12	13521
Supramarginal gyrus	L	48	5.23	−63	−24	21	
Supramarginal gyrus	R	40	4.97	60	−36	36	
Superior parietal lobule	L	5	4.95	−21	−48	69	
Supplementary motor area	L	32	4.94	0	9	51	
Cingulate cortex	L		4.92	−9	−6	42	
Rolandic operculum	L	48	4.92	−54	6	3	
Insula	L	48	4.79	−42	−3	0	
Insula	R	47	4.76	39	24	3	
Superior temporal gyrus	R	42	4.71	60	−39	18	
Postcentral gyrus	L	48	3.87	−54	−18	17	
Putamen	R	48	3.76	31	13	8	
Putamen	L	48	3.62	−31	−10	5	
Frontal gyrus	R	6	4.41	42	3	60	125
Cerebellum	R		4.32	24	−54	−57	2695

L, left; R, right.

**Table 3:** Activation in response to heat stimulation in the PD group.

Region	PD group						
	Laterality	BA	*Z*	MNI coordinates (mm)			Cluster
			Score	*x*	*y*	*z*	(Voxels)
Supramarginal gyrus	R	40	4.08	66	−33	27	10648
Superior parietal lobule	R	7	3.98	30	−57	60	
Supramarginal gyrus	L	40	3.94	−63	−42	36	
Hippocampus	R	20	3.77	33	−21	−9	
Rolandic operculum	L	48	3.51	−46	−21	22	
Insula	L	48	3.12	−38	−18	23	
Insula	R	48	2.99	35	−18	24	
Postcentral gyrus	L	48	2.82	−58	−16	22	
Superior temporal gyrus	L	38	2.75	−53	5	−1	
Cerebellum	L		3.53	−14	−78	−25	412

L, left; R, right.

**Table 4:** Decreased activation induced by heat stimulation in patients with PD in contrast to healthy controls.

Region	PD versus control group						
	Laterality	BA	*Z*	MNI coordinates (mm)			Cluster
			Score	*x*	*y*	*z*	(Voxels)
Superior temporal gyrus	L	22	4.51	−63	−27	12	485
Insula	L	47	2.94	−39	18	−6	
Middle temporal gyrus	R		3.34	60	−48	15	36
Temporal pole	R	38	3.30	54	6	−12	120
Superior temporal gyrus	R	48	3.25	54	−9	3	
Insula	R	48	2.91	48	15	−6	

**Table 5:** Correlation between mean *β* value and VAS and HPT.

		VAS			HPT		
		CON	PD	CON + PD	CON	PD	CON + PD
STG.L	Correlation coefficient	0.510	−0.236	0.439	−0.53	−0.19	−0.439
	*P* value	0.036^*^	0.417	0.013^*^	0.028^*^	0.515	0.013^*^

STG.R	Correlation coefficient	0.489	−0.045	0.450	−0.71	0.003	−0.473
	*P* value	0.046^*^	0.879	0.011^*^	0.001^*^	0.991	0.007^**^

INS.R	Correlation coefficient	0.560	0.500	0.630	−0.56	0.184	−0.303
	*P* value	0.019^*^	0.068	0.000^**^	0.019^*^	0.528	0.097

Note: ^*^ *P* < 0.05; ^**^ *P* < 0.01.
